# Importance of the actual plant height in modulating the within-community spectrum of plant form and function

**DOI:** 10.3389/fpls.2025.1616656

**Published:** 2025-07-24

**Authors:** Dong He, En-Rong Yan, Li-Ting Zheng, Yan-Jun Song, Xiao-Dong Yang, Wen-Hui You, J. Hans C. Cornelissen

**Affiliations:** ^1^ College of Ecology and the Environment, Xinjiang University, Urumchi, China; ^2^ Shanghai Key Lab for Urban Ecological Processes and Eco-Restoration, School of Ecological and Environmental Sciences, East China Normal University, Shanghai, China; ^3^ Systems Ecology, Department of Ecological Science, Faculty of Science, Vrije Universiteit (VU University), Amsterdam, Netherlands

**Keywords:** crown architecture, dry-mass allocation allometry, hydraulic limitation, leaf and wood economics, local scale, maximum plant height, plant ecological strategies

## Abstract

Maximum height (H_max_) is a principal driver or correlate of interspecific variation in many plant functional traits. Still, it remains unclear why leaf resource economic traits are invariant with H_max_ at global scale and why broad-scale interspecific trait correlations are not retained at local scale. Here we proposed that the actual plant height (H_act_), which is tightly linked with highly localized abiotic and biotic interactions, is more important than H_max_ in determining plant morpho-physiological traits among locally co-occurring plants. We tested the idea across community, regional, and global scales. We also examined correlations among 22 traits, including leaf physiology, hydraulics, and crown architecture, within a subtropical forest in Eastern China. Additionally, we explored how H_act_-driven trait variations align with vertical patterns of microclimates. Results showed stronger correlations between leaf traits and H_act_ at the community level, except for leaf area. Intraspecific variation exceeded interspecific variation, and trait correlations were stronger at the individual level than at the species level. H_act_ positively correlated with traits like crown area, leaf mass per area, stomatal density, and hydraulic conductivity but negatively with stem hydraulic safety margin and leaf coverage. Vertical changes in photosynthetically active radiation explained most H_act_-driven trait variations. Our findings suggest that H_act_ influences plant trade-offs in biomass allocation and photosynthetic-hydraulic limitations, shaping functional diversity within communities. This highlights H_act_ as a key factor in balancing resource use, support, and water transport among coexisting plants.

## 1 Introduction

Height is one of the most important determinants of plant form and function as it represents access to sunlight, and competitive vigor via woody structures and mechanical strength (Westoby, 1998; Diaz et al., 2016). Maximum height (H_max_), defined as the upper limit of height a plant species can attain, has been recognized as a key axis of ecological strategic differentiation. Growing evidence has revealed that interspecific differences in H_max_ are associated with distinct morpho-physiological traits (King, 1990; Reich, 2000; Falster and Westoby, 2005; Cavaleri et al., 2010; Givnish et al., 2014). However, recent global scale surveys have shown that the “leaf economic spectrum” (LES, *sensu* Wright et al., 2004), i.e. traits linked to carbon and nutritional investments in photo-assimilation capacity versus resource protection, are either invariant (Price et al., 2014) or weakly correlated with H_max_ (Diaz et al., 2016). In addition, the global LES does not hold up among species at local scale (Hulshof and Swenson, 2010; Wright and Sutton-Grier, 2012; Messier et al., 2017a, 2017b; Bruelheide et al., 2018). The weak H_max_-trait relationships at broad scale and the absent LES at local scales, therefore, call for two basic questions—i) why is H_max_ so weak in capturing variations in leaf economic strategies? and ii) does the actual plant height (H_act_) as measured in a given community play an important role in modulating the local leaf or whole-plant economics spectrum?

An important aspect of the plant economics spectrum is the correlation of the leaf and wood traits across species, which reflects the degree to which different sets of plant functional strategies co-vary with one another (Wright et al., 2004; Chave et al., 2009; Reich, 2014). Trait correlation patterns describe how species strategies are shaped by strong selection along the main axes of trait trade-offs (Poorter et al., 2014). In tandem with coupling all resources, strong selection along trait trade-off axes can cause species to unify ecological strategy across all plant organs (Reich, 2014). Strong trait correlations identified at global scale suggest that natural selection shapes the interdependence of the plant form and function over broad spatial scales. However, at local scales, the strength of trait correlation is determined by the co-occurring plants that vary in H_act_ and respond individually to vertical variation in microclimate. For instance, plant height can cause substantial variations in leaf and wood economic traits, sap flow and photosynthetic rates (Ryan and Yoder, 1997; Herault et al., 2011; Martin and Thomas, 2013; Sendall and Reich, 2013; Kenzo et al., 2015; Damian et al., 2018). Consequently, H_act_ is a principal driver of variation in both singular traits and trait-trait relationships in local communities (Falster and Westoby, 2005; He and Yan, 2018).

H_max_ is biologically meaningful for capturing trait variations at broad spatial scales, where a remarkable diversity of plant forms and life histories potentially shapes the wide spectrum of plant form and function (Diaz et al., 2016). However, interspecific differences in H_max_ are generally smaller within than across communities, due to lower species and functional diversity at local than at regional scales (Marks et al., 2016). Therefore, small variation in H_max_ at within-community scale might not keep pace with the great variations in plant physiological and hydraulic properties across differently sized individuals within and among species. In addition, H_max_ of a species at the within-community scale may be weakly indicative of the vertical variation in microclimate such as light intensity, which is a key driver of trait variation across forest strata (Cavaleri et al., 2010). In contrast, H_act_, which is measured for actual entire individuals within a community, can accurately quantify overall patterns of plant form and function. In fact, individuals within a species are not uniform but plastic in their life history strategies (Anderegg et al., 2018). In local communities, within-species trait plasticity can contribute much to the intraspecific plant economics spectrum (He and Yan, 2018). As a crucial determinant of the fine-scale variations in plant physiological and hydraulic functions, H_act_ thereby serves as a leading dimension capturing a large proportion of trait variances across co-occurring individuals within communities (Onoda et al., 2014; Letten et al., 2015; Rungwattana and Hietz, 2018).

Here we critically examined whether variation in H_act_ across locally co-occurring woody plants modulated the spectrum of plant form and function revealed at global scale. We explored variation and covariation of three groups of traits with H_act_: leaf and wood economic and physiological traits, leaf and wood hydraulic traits and crown architectural traits (**Table 1**). These trait groups are involved in the leaf economics spectrum (LES), the wood economics spectrum (WES) while, across plant organs, allometric rules driving leaf-stem coordination related to plant hydraulics also apply (Anfodillo et al., 2006; Zhong et al., 2019; Zhong et al., 2020). Together these trait groups are thought to represent trade-offs and coordination among physiological and biomechanical functions between species (Messier et al., 2017a). The LES reflects a trade-off between resource acquisition and conservation (Wright et al., 2004), and the WES reflects trade-offs among transport safety, transport efficiency and mechanical support (Chave et al., 2009). Crown architectural traits reflect how selection shapes plant forms to optimize the relationships among sap transport, light harvesting and mechanical support (Iwasa et al.; Ellsworth and Reich, 1993; Smith and Sperry, 2014). Specifically, we tested how plant traits associated with different physiological and biomechanical functions vary with the H_act_ at within-community scale, and to what extent these relationships differ from the global surveys. In addition, we examined whether LES, WES and crown architectural traits were interdependent from each other, and whether trait correlations were stronger across individuals than across species at within-community scale. Moreover, we tested how vertical change of microclimate associated with trait variation across co-occurring individuals. We hypothesize that leaf and wood economics traits and crown architectural traits co-vary with H_act_ across individuals at within-community scale (**Table 1**). We expect that the strength of the H_act_-trait relationship is stronger than the H_max_-trait relationship across spatial scales. Further, we hypothesize that the correlation strength among leaf and wood economic traits and crown architectural traits is stronger at individual than at species levels, as a result of the individual-based multiple proximate physiological and biomechanical trade-offs and coordination among locally co-occurring plants (Reich, 2014). Moreover, we predict that vertical changes of light availability and air humidity are associated with variability of plant leaf and wood economic traits, light-relevant physiology traits, hydraulic and crown architectural traits at within-community scale.

**Table 1:** The 22 study traits (Acronym) and the corresponding morphological and physiological trade-offs involving shifts with plant height in resource acquisition/use strategy, photosynthetic hydraulic limitation and biomass/size allometry.

Organ	Trait (Acronym)	Hypothesized shift with plant height			Refs
		Resource acquisition (+) or conservation (-)	Hydraulic efficiency (+) or safety (-)	Positive (+) or negative (-) allometry	
Leaf	Leaf area (LA)	+	+	+	1:3, 10
	Leaf mass per area (LMA)	+	–	+	1:13
	Leaf nitrogen content (Nmass)	+	#	#	1:4, 8:16
	Leaf dry matter content (LDMC)	+	#	#	3, 15
	Maximum photosynthetic rate (Amax)	+	+	#	2, 4:10, 16:18
	Light compensation point (Lcp)	+	#	#	9, 10
	Stomatal density (SD)	+	+	#	absent
	Leaf water potential (Lwp)	+	+	#	10, 17:19, 22, 23
	Stomatal conductance (Cond)	+	+	#	10, 13, 17, 19, 20
	Transpiration rate (Tr)	+	+	#	10, 18
	Petiole diameter (Pd)	+	+	+	11, 22
Branch	Specific hydraulic conductivity (Ks)	+	+	#	3, 17, 19, 25
	Xylem vessel diameter (Vd)	+	+	+	23, 25
	Wood density (WD)	#	–	+	1:4, 17, 19, 25
Stem	Hydraulic safety margin (SM)	–	–	#	12, 17, 22, 23, 25
	Sap flow flux (E)	+	+	#	20, 21
Crown	Proportion dispersed leaves (Ld)	–	–	#	26, 27
	Leaf coverage (Lcov)	+	–	#	26:29
	Crown area (Ca)	+	+	+	27:29
	Light exposure index (Lex)	+	#	#	26, 27
Whole plant	Actual height (H_act_)				
	Maximum height (H_max_)				

The signs of plus (+) or minus (-) indicate the hypothesized shift in direction of each trait with plant height and the number sign (#) indicates uncertain direction or irrelevant functions.

To test the above hypotheses, we examined both interspecific and intraspecific correlations of 22 traits associated with physiological and biomechanical functions of woody plants (**Table 1**), across 60 individuals of 19 species coexisting within a subtropical forest community. Additionally, five common traits for 475 individuals of those 19 species at regional scale were also involved in the analysis. We initially examined the bivariate trait relationships at both within-community and regional scale. Second, we ran principal component analysis (PCA) to demonstrate the within-community spectrum of plant form and function. Finally, a simple linear regression was used to test how trait and trait dimension vary with vertical change of microclimate.

## 2 Materials and methods

### 2.1 Study site

This study was conducted in Tiantong National Forest Park (29°41-50′N, 121°36-52′E), situated in the Eastern Zhejiang Province, China. This area is subject to a subtropical monsoon climate, with a hot, humid summer and a drier cold winter. The annual mean temperature is 16.2°C, the warmest month is July with a mean temperature of 28.1°C, and the coldest is January with a mean temperature of 4.2°C. Annual precipitation is 1374.7 mm, with a majority concentrated from May-August. The soils of this area are mainly krasnozem and zheltozem, with pH values ranging from 4.4 to 5.1 (Yan et al., 2009). The largest portion of the vegetation in the Park is represented by *Schima superba* dominated forest communities, which are sub-climax evergreen broadleaved forests and also widespread across mountains and low hills in subtropical China.

### 2.2 Sampling strategy

In the interior of the Park, a *Schima superba* dominated forest plot with an area of 800 m^2^ was specifically selected in order to examine how differences in H_act_ among co-occurring woody plants affect trait variation and trait correlations across vertical layers where microclimate shifts gradually within the community. The horizontal environmental heterogeneity in the plot is fairly low. The slope inclination is about 10° (facing east), and the elevation of the plot is 120 m above sea level. Soil moisture within the plot was about 16% in the dry and 29% in the wet season from 2014 to 2015. Total nitrogen and phosphorus concentration is respectively about 2.99 ± 0.10 and 0.11 ± 0.05 mg g^-1^ (unpublished data), indicating a high level of phosphorus limitation. The community structure has two apparent vertical layers: overstorey and understorey, with H_act_ ranging from 1.8 m to 18 m for woody plants. Stem density is 6400 individuals per hectare, and there are 60 individuals of 19 species with H_act_ larger than 1.5m in the plot. The overstorey is dominated by *Schima superba* while *Castanopsis fargesii*, *Cyclobalanopsis myrsinifolia*, and *Lithocarpus glaber* are subordinate. In the understory, *Camellia fraterna*, *Eurya loquaiana*, *Rhododendron ovatum*, and *Symplocos sumuntia* are co-dominant species. The details for the characteristics of these species and their constituent individuals are provided in the Supporting Information (**Supplementary Table 1**).

We measured 22 traits across 60 woody plants within the *Schima superba* community. For singleton and doubleton species within the plot, we sampled only those one or two individuals. In doing so, we ensured that all species nested within plot had their trait measurements *in situ* and thus accounting for the local field reality of predominant contributions of some abundant species to intraspecific variations and relatively small contribution of rare species. Twenty-two traits were selected mainly for those generally measured in the LES, WES, sap transport and plant crown architecture that associate directly with important physiological, hydraulic and biomechanical functions in plants (see **Table 1** for details).

Further, to detect whether the H_act_-based trait variations and trait-trait correlations at within-community scale differ from those H_max_-based patterns at regional scale, we also measured 5 traits on 475 individuals from the same 19 species across 5 sites in the Ningbo region (see trait measurement). These five sites were spaced approximately 25 km from each other and had been protected from logging and clearcutting for at least 30 yr. Consequently, the vegetation in those five sites resembles semi-mature forest by sharing broadly similar species composition with the studied plot in the Park. In each site, five healthy individuals per species were selected within intact forests for the measurement of plant traits in August, 2015. Since our aim was to screen trait variation at regional scale, we focused on the mean trait values for a given species in a specific site and thus only mature individuals were selected, by following the standard protocols (Perez-Harguindeguy et al., 2013). In this case, 5 traits from the leading trait dimensions (cf. **Table 1**) were specifically measured, due to the difficulty of collecting all traits such as stem sap flux over large spatial scale in the same time.

### 2.3 Trait measurement

Following standardized protocols (Cornelissen et al., 2003), we measured mean leaf area (LA), leaf mass per area (LMA), leaf dry matter content (LDMC), leaf nitrogen content (Nmass) and wood density (WD) at both the *Schima superba* plot in the Park and the five sites at regional scale. These five traits were measured in the field or in the laboratory (the specific methods detailed in Appendix S1). Also, the value of H_max_ for 19 species was retrieved from the *Flora Republicae Popularis Sinicae* (http://foc.eflora.cn).

In addition to the above five traits, 16 out of the 22 traits were specifically measured on the co-occurring individuals within the *Schima superba* plot in the Park during the plant growing season from July to September in each of 2014 and 2015. For each individual, we measured architectural traits for H_act_, projected crown area (Ca), leaf coverage (Lcov), leaf convergence and petiole diameter (Pd). Lcov was estimated with a scale of 10% cover increments. Leaf convergence was classified into clumped and dispersed grouping, respectively. Here we used the proportional dispersed leaves per crown size (Ld, opposite to the proportional clumped leaves by assuming that the proportion of dispersed plus clumped leaves is 100%) to quantitatively describe how plants acclimatize to light conditions and hydraulic restrictions in their leaf deployment *per se* (Poorter et al., 2006). Although Pd is highly associated with plant physiological and hydraulic functions, we grouped it into architectural trait dimensions due to its morphological nature to support leaves.

Light-relevant morphological and leaf physiological and hydraulic traits were measured, such as light compensation point (Lcp), the maximum photosynthetic rate (Amax), transpiration rate (Tr), stomatal conductance (Cond) and stomatal density (SD) in leaves. Furthermore, Crown light exposure index (Lex) was determined on a five-point scale for each plant: 1 = no direct light received in the crown area, 2 = lateral light received in the crown area, 3 = partial overhead light received in the crown area, 4 = more than 90% of the crown area receives direct overhead light, and 5 = emergent crown with direct light from all direction (Poorter et al., 2006). For each plant, one branch in the peripheral position (sunlit-side) of the crown was cut down and quickly stored in a water filled bucket on the field. In order to avoid effects of blight or pest attack, three healthy leaves per branch were selected for leaf photosynthetic and transpiration measurements between 8:00 am and 4:00 pm on each sunny day with a portable photosynthesis system (Li-6400XT, Li-Cor, USA). The remaining healthy leaves on the collected branches were also detached and kept in a refrigerator under 4°C for the measurement of SD.

In addition, we measured hydraulic and wood anatomical traits for leaf water potential (Lwp), sapwood specific hydraulic conductivity (Ks), stem hydraulic safety margin (SM), xylem vessel diameter (Vd) and sap flow flux (E) for each individual. Three branches were harvested from the sun-exposed position of the plant crown before the sunrise (i.e., predawn) and sealed in a black plastic bag with moist towel, and immediately transported to the laboratory within 15 min to measure Lwp by using a pressure chamber (Model 1505D-EXP, PMS Instrument Company, Albany, OR, USA). We collected 1-year-old twigs (3-6 mm in diameter) to measure Ks with a high-pressure flow meter (HPFM-Gen3; Dynamax, USA). SM was estimated by measuring percent loss of hydraulic conductivity of twigs for each sampled branch under different stem xylem pressures with the air injection method (Cochard et al., 1992). Vd was measured for the same twigs. Vessel lumen area and vessel density were determined from transverse twig sections by using a microscope (Olympus DP73, Japan) fitted with a digital camera (QColor 3; Qimaging, Burnaby, BC, Canada). Vessel lumen areas were averaged to generate individual means and Vd was calculated from the diameter of a circle of the given lumen area. Stem sap flow was monitored by using two FLGS-TDP XM1000 systems (Dynamax Inc., Houston, TX, USA) during one year from July 2014 to July 2015, and subsequently the maximum of E in summer was used to characterize variation in water transport capacity for each plant. The specific methods for the measurement of these traits are provided in the Supporting Information (**Supplementary Text 1** and **Supplementary Table A1**).

### 2.4 Microclimate measurement

We mounted a steel tower for a height of 20 m within the center of the studied plot for monitoring the long-term vertical variations in microclimate within the studied *Schima superba* plot. Air humidity, temperature and photosynthetic active radiation were measured in four vertical layers over a one-year period from July 2014 to July 2015. Across four vertical locations, a microclimate monitoring system (EM50, Decagon, USA) was equipped independently. At the height of 2.5 m, 7.5 m, 11.5 m and 16.5 m, a QSO-S photosynthetic active radiation (PAR) photon flux sensor, a VP-3 humidity temperature sensor and a thermometer were installed for monitoring photosynthetic active radiation, air humidity and temperature, respectively. All these sensors collected data at 30-min intervals.

We calculated annual means of microclimate variables for each vertical layer. Variations in microclimate were also calculated diurnally, due to the great differences between day and night time. According to the sun-set date of the growing season in the studied site, we divided a day into four stages: morning (7:00 to 10:59 am), noon (11:00 am to 14:59 pm), afternoon (15:00 to 17:59 pm), and night (18:00 pm to 6:59 am). In the subsequent analyses, we used the noon day means of photosynthetic active radiation, air humidity and temperature to represent vertical patterns of these microclimate factors.

### 2.5 Data analysis

We used a linear mixed model to decompose trait variation across and within species to demonstrate how each trait varies intra- and inter-specifically at within-community scale. Since the mixed model assumes that the observations within each subgroup are normally distributed and have equal variances, we log_10_-transformed the data for each of the 22 plant traits to achieve the normality of both residuals and random effects in the calibrated linear model. This analysis was conducted using a restricted maximum likelihood (REML) method, and variance components were extracted with the ‘varcomp’ function in R package ‘ape’.

To test whether the plant height-functional trait relationships apparent in global interspecific surveys still holds at the small spatial scale, the bivariate trait relationships were examined separately for locally co-occurring plants at both individual and species levels, and for the same species across the studied region, by using Pearson correlation analyses. For species level analysis, the mean value for each trait was employed to relate with H_max_. We specifically compared the strength of the correlations between H_act_ and traits across individuals at within-community scale differed from those between H_max_ and traits presented at each of within-community, region and global scales. The global correlation coefficients in the relationship between H_max_ and traits were combined from (Price et al., 2014) and (Diaz et al., 2016). In addition to relationships with plant height, SMA regression was conducted again for testing the bivariate trait relationships across other leaf and wood economics traits, leaf physiological traits, hydraulic traits and architectural traits of local co-occurring plants at both individual and species levels.

Moreover, we conducted a principal component analysis (PCA) for all traits across individuals in order to test how traits that represent different functional dimensions were interdependent, thereby composing the within-community spectrum of plant form and function. Given the generally high proportions of variance explained by the first two axes (PC1 and PC2), these scores were used in subsequent analyses as a proxy for the key trait dimensions. To understand the patterns of variation between individual trait and trait dimensions, the scores of PC1 and PC2 were also correlated with each trait.

Finally, to test whether H_act_-related trait variations were impacted by the vertical variation in microclimate, a linear regression was conducted for each plant trait and key trait dimensions (i.e., PC1 and PC2 in the PCA) against each of air humidity and photosynthetic active radiation. All statistical analyses were realized in R (R Core Team, 2017), with the aid of the R packages, including ‘smatr’, “ade4”,’varcomp’, and ‘lmerTest’.

## 3 Results

### 3.1 Inter- and intra-specific trait variations

**Figure 1:**
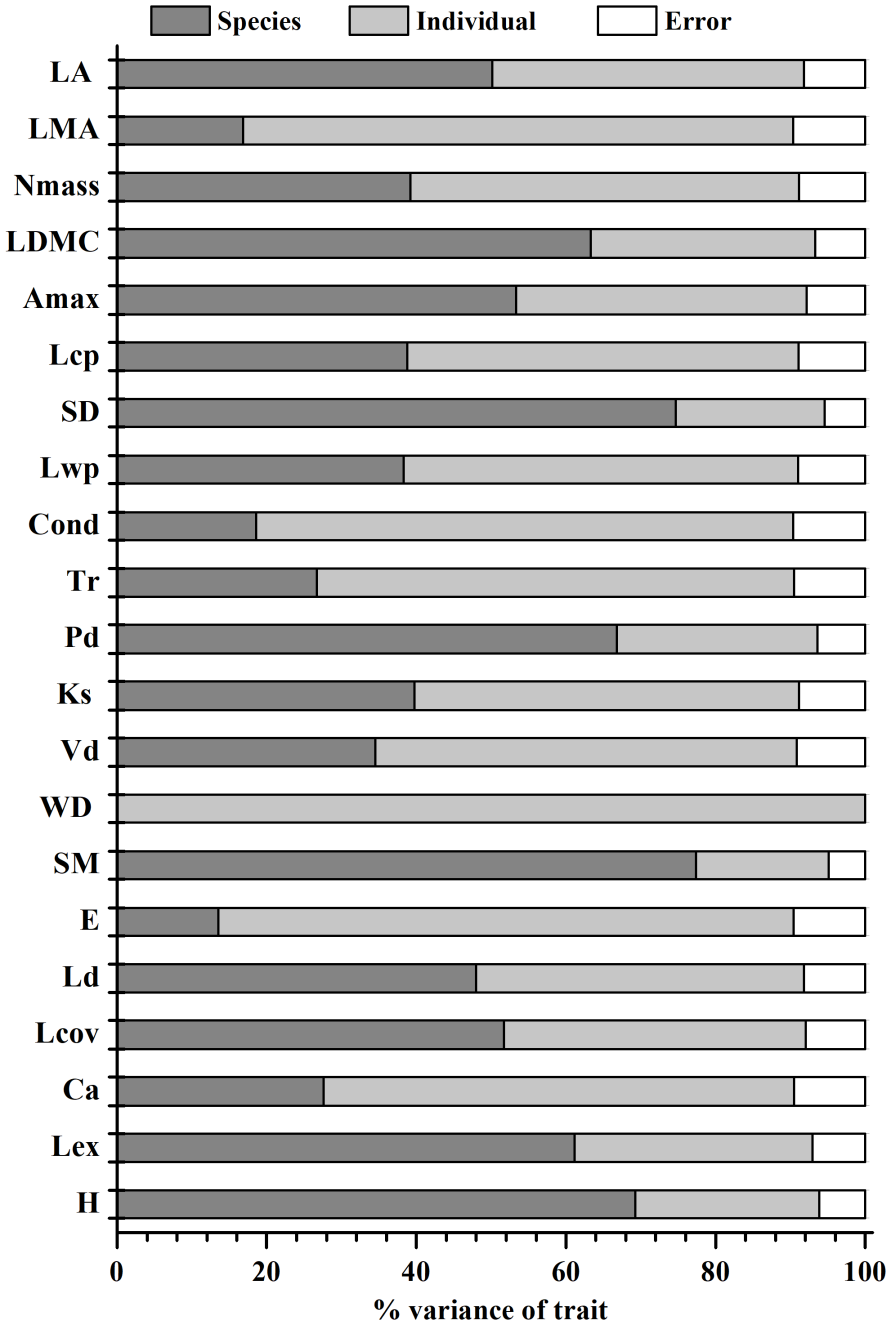
Variance partitioning of the leaf and wood economics traits, leaf physiological traits, hydraulic traits and plant architectural traits among and within woody species. See **Table 1** for trait acronyms.

Variation in leaf economics and physiological traits, and hydraulic and architectural traits showed distinct patterns among and within species (**Figure 1**). For leaf economic and physiological traits, 52%, 74% and 53% of total trait variance was attributed to within-species level for Nmass, LMA and Lcp, respectively, while 50%, 63% and 53% of total variance was at the interspecific level for each of LA, LDMC and Amax. For most hydraulic traits, the within-species level was responsible for more than 50% of total trait variation, the only exception being SM (18%). Remarkably, 99% of overall variance in WD was due to within-species variance. For architectural traits, variance in Ld, Lcov, Pd and plant height were accounted for mainly by species level, but variance in Ca was attributable mostly to within-species level (63%).

### 3.2 Trait correlations at individual and species levels

**Figure 2:**
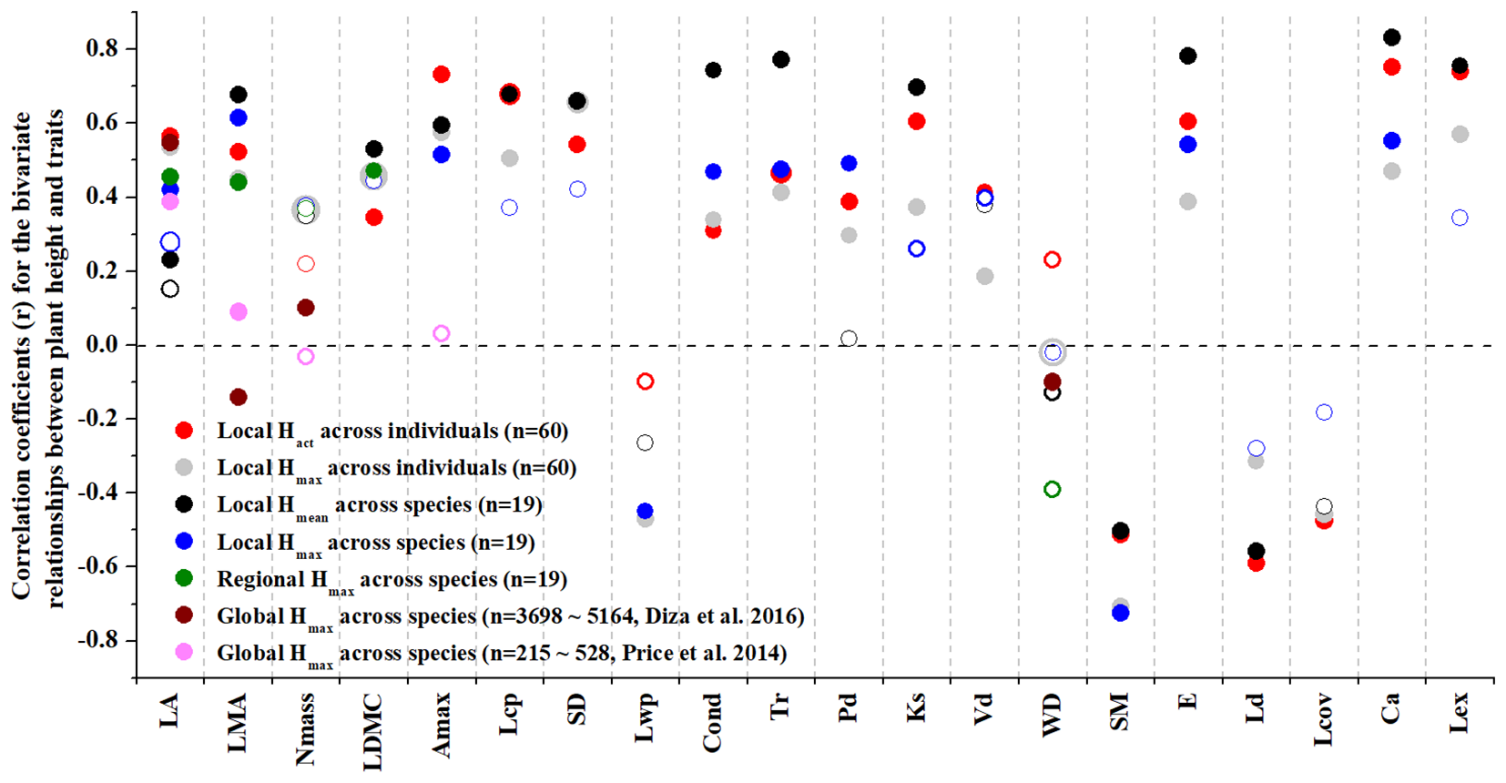
The coefficients (r) of Pearson correlation for the bivariate relationships between plant height and each of the leaf and wood economics, physiological and hydraulic traits and crown architectural traits across woody species and/or across woody individuals at within-community, regional and global scales as revealed by standardized major axis (SMA) regression. Note that, in the SMA regressions, H_act_ and H_max_ are used separately across individuals, H_mean_ (species mean) and H_max_ are used separately across species at local scale, and H_max_ is employed only at regional and global scales. The global scale results were obtained from both Diaz et al. (2016) and Price et al. (2014). Open circles indicate that the regression relationship is not significant. See **Table 1** for trait acronyms.

The bivariate relationships between plant height and leaf economic traits were stronger at within-community and regional scales than at global scale; only the H_max_-leaf area relationship at global scale showed similar correlation strength as revealed at within-community and regional scales (**Figure 2**). Plant height did not significantly correlate with wood density at within-community and regional scales, compared to their negative correlation at global scale. At within-community scale, relative to the H_max_, the actual plant height being assigned at individual level (i.e., H_act_) and averaged at species level (i.e., H_mean_) explained more of the variations in most leaf economics traits, light-relevant physiological traits, water transport traits and crown architectural traits. However, H_max_ explained more of the species-level variations in LMA, SM, Lwp and Pd (**Figure 2**).

**Figure 3:**
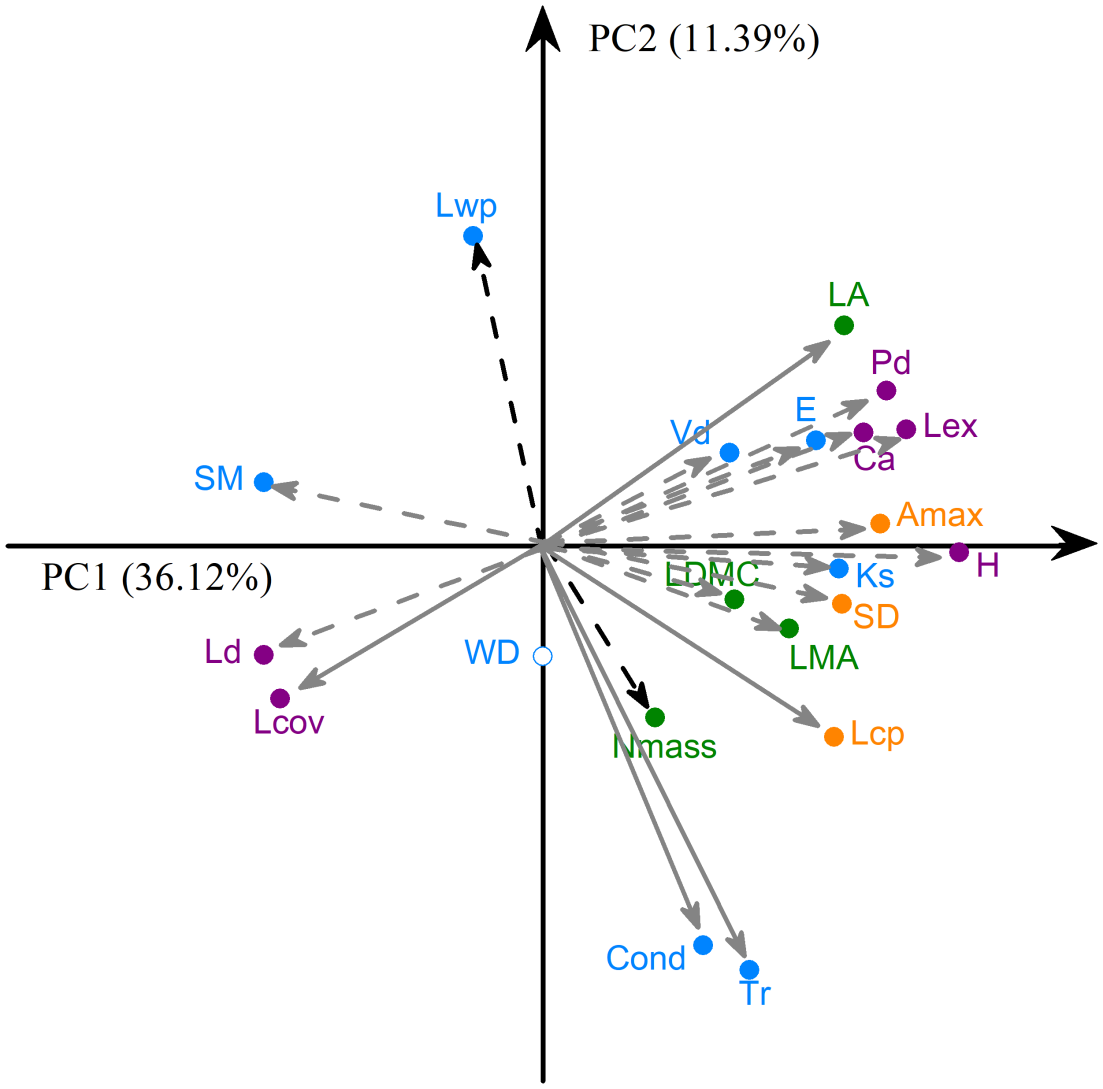
Principal component analysis on the 21 traits across 60 co-occurring woody individuals at within-community scale. Lines show how each trait is correlated with the first two axes (loadings). Traits significantly correlated with each of the PC1 and PC2 are shown with grey and black dashed lines, respectively, while those significantly correlated with both axes are shown with grey solid lines. See **Table 1** for trait acronyms.

The correlation strength between plant height, leaf and wood economic, physiological and hydraulic traits and crown architectural traits was generally more pronounced across individuals than across species at within-community scale. H_act_ was positively correlated with LA, LMA, LDMC, Amax, Lcp, Lex, SD, Vd, E, Ks, Cond, Tr, Ca and Pd and negatively correlated with SM, Ld and Lov at individual level, but H_max_ was only positively correlated with LMA, Amax, E, Cond, Tr, Ca and Pd and negatively correlated with SM at species level (**Figure 3**; **Supplementary Figure 1**).

Principal component analysis showed a pronounced covariation among leaf and wood economic and physiological traits, among leaf and wood hydraulic traits, and among crown architectural traits across co-occurring woody individuals at within-community scale, and the percentages of variation in plant traits explained by PC1 and PC2 were 36.1% and 11.4% (**Figure 3**). Along the PC1 axis, with the increase of plant height, LA, LMA, Amax, Lex, SD, Lcp, E, Vd, Ks, Tr, Cond, Pd, Ca increased, while SM, Ld and Lcov decreased. The PC2 axis was positively loaded by Lwp and LA while negatively loaded by Lcp, Nmass, Tr, Cond and Lcov.

### 3.3 Relationships between plant traits and microclimate across community vertical layers

**Figure 4:**
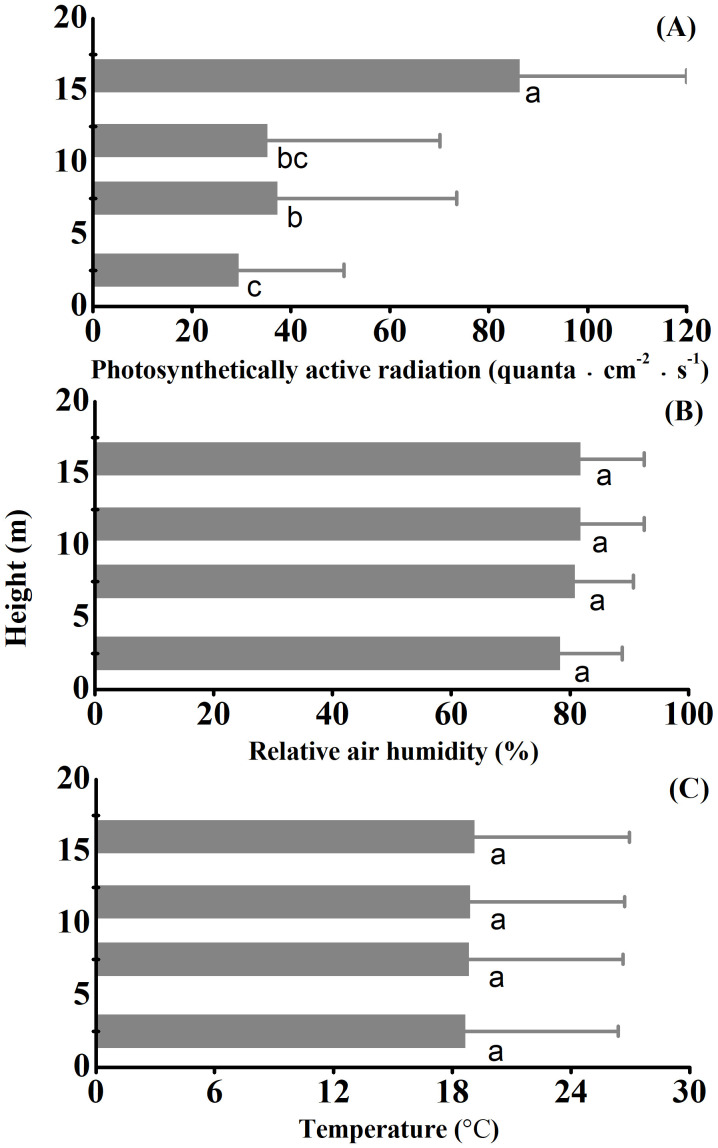
Variation in photosynthetically active radiation **(A)**, relative air humidity **(B)** and temperature **(C)** across strata of the studied *Schima superba* forest community. The three microclimate properties were monitored in four strata across 365 days. The error bars onto the barplots are indicative of temporal variation rather than of sampling errors over the vertical gradient. Different letters next to bars denote significant differences through Fisher’s least significant difference (LSD) tests.

**Figure 5:**
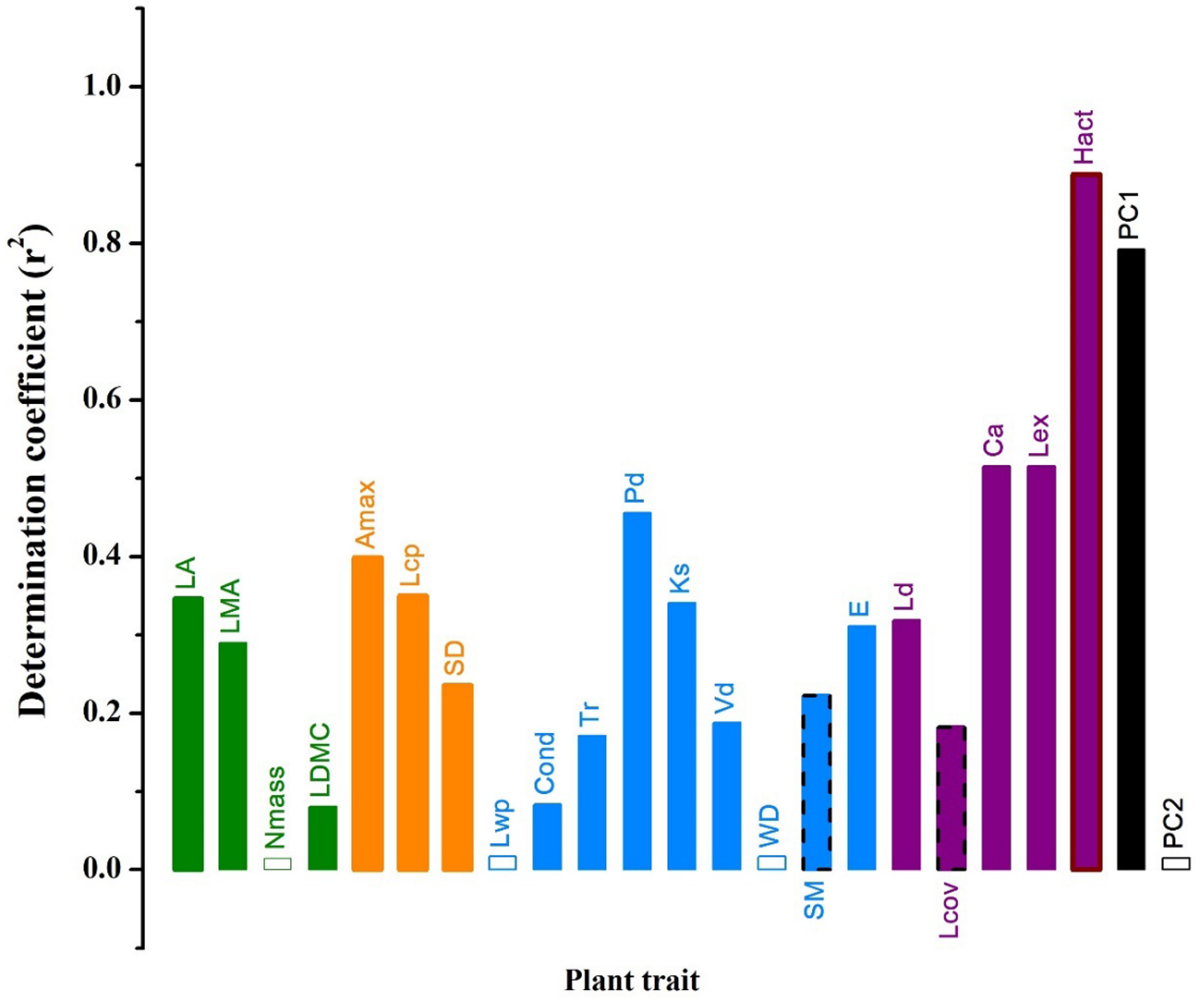
The determination coefficients of the linear relationships between plant traits of co-occurring woody individuals and photosynthetically active radiation across four strata within the studied *Schima superba* forest forest community. The solid bars with and without border indicate significant negative and positive linear relationships, respectively, while the hollow bars indicate the lack of significant linear relationships between plant traits and microclimate properties. See **Table 1** for trait acronyms.

Photosynthetically active radiation significantly increased while relative air humidity or temperature did not vary across strata overall (**Figure 4**). Except for Nmass, WD, Lwp and PC2 scores, all other traits and PC1 scores increased while SM and Lcov significantly decreased with photosynthetically active radiation (**Figure 5**).

## 4 Discussion

### 4.1 Linkages between multiple plant traits and actual versus maximum plant height

This study explicitly addresses whether actual plant height (H_act_) is more effective than maximum plant height (H_max_) for capturing trait variations and modulating trait correlations among locally co-occurring individual woody plants. We found that traits representing distinct functional strategies associated more strongly with H_act_ than with H_max_, and that correlations among leaf economic and physiological traits, hydraulic and crown architectural traits were stronger at individual than at species levels. In addition, we found that vertical patterns of light availability could explain most H_act_-related trait variations at within-community scale. These results highlight the importance of the actual plant height for regulating both horizontal (between individuals and species) and vertical patterns of morpho-physiological leaf and wood traits and canopy attributes of locally co-occurring plants; i.e. for the 3-D trait structure of the forest. This study thereby provides strong evidence that the actual plant height plays a non-negligible role for structuring the within-community spectrum of plant form and function.

Over the last three decades, the growing interests in plant height-related trait variations have greatly advanced our understanding of how trees that characteristically differ in H_max_ have similar or different phenotypic attributes (King, 1990; Reich, 2000; Falster and Westoby, 2005; Meinzer et al., 2011). H_max_ is also an important part of a coordinated suite of life-history traits including seed mass, time to reproduction, longevity and seed production per year (Westoby, 1998; Diaz et al., 2016). Despite the fundamental role of crown light exposure index (Lex) for indicating interspecific differences of plants’ light adaptive capacity and reproductive biology, as well as potential competitive vigour, it is difficult to link H_max_ with functional differences of leaf economic traits across competing individuals (especially for those individuals within-species), due to leaf economic traits being highly dynamic variables that depend greatly on the ontogenetical stage of the plant (Garnier et al., 2016). However, among previous empirical studies regarding the spectrum of plant form and function, leaf economic traits have often been measured at a given ontogenetic stage only, such as the adult stage (Price et al., 2014; Diaz et al., 2016) or the sapling stage (Messier et al., 2017a). In such studies, the failure to include H_act_-dependent trait variation in the spectrum of plant form and function may be one of the reasons why H_max_ is weakly indicative of the leaf economic spectrum across broad and local spatial scales (Price et al., 2014; Diaz et al., 2016). Fundamentally, our results support this argument by revealing how more of the variation in leaf economics traits could be explained by H_act_ than by H_max_ at within-community scale (**Figure 2**).

The H_act_-dependent trait variations might also partly cause the differences between local and global patterns of trait correlations. Recent studies suggest that the relationships between leaf economics traits identified primarily at global scales are not retained at local scales (Hulshof and Swenson, 2010; Wright and Sutton-Grier, 2012; Funk and Cornwell, 2013; Messier et al., 2017b). For example, the correlation coefficient (i.e., r^2^) between SLA and Nmass across a global sample of species is about 0.57 (Wright et al., 2004). However, within sites or small regions, such as in this study, the r^2^ is often much lower than this (**Figure 2**), or absent altogether (Wright and Sutton-Grier, 2012; Messier et al., 2017a). In our study the within-species scale was responsible for about half of the total trait variance in most of the leaf economic traits investigated (**Figure 1**). This relative large intraspecific trait variation indicates a large degree of within-species phenotypic plasticity and phenotypic response to actual abiotic variability, which can substantially alter the strength and even the direction of the leaf economics spectrum at local scales (Anderegg et al., 2018). Therefore, the unsubstantial interspecific correlations among leaf economic traits at local scale might be a result of ignoring H_act_-dependent trait variation among co-occurring individuals within species.

Architectural traits play a central role in linking cross-species patterns of resource acquisition, mechanical support and hydraulic functions (Poorter et al., 2006; Messier et al., 2017a). Our results demonstrate further that, compared to species level, individual level crown architecture associates more strongly with the physiological, hydraulic and biomechanical functions across leaves, branches and stems at local scales (**Figures 3**, **4**). The observed coupling relationships among leaf economic and physiological traits, hydraulic traits and architectural traits (**Figure 3**) indicate that there is a strong network of intraspecific trait associations among locally co-occurring individual plants, while such associations are relatively weak among species at local scale (**Supplementary Figure 1**). This again calls for attention to the non-negligible role of the H_act_ in imposing biophysical and environmental constraints on strategic trade-offs among locally co-occurring plants. H_act_-mediated individual trait variation should accurately capture species’ plasticity and their actual phenotypical responses to local microclimate variability (Cianciaruso et al., 2009). The clear individual-level trait correlations in this study, therefore, support the perspective that the plant economics spectrum operates at the whole-plant scale (Reich, 2014; Diaz et al., 2016). Robust trait correlations at individual level suggest that localized abiotic and biotic selections can shape the phenotypic space and the interdependence among distinct trait dimensions, thus modulating the within-community spectrum of plant form and function.

### 4.2 Direction and strength of covariation of different traits with H_act_

Correlation of traits is essential to the ecology and evolution of diversity in plant form and function, since it reflects how plant strategies are shaped by strong selection along the main axes of trait trade-offs due to environmental and biophysical constraints (Poorter et al., 2014). As an estimator of the plant’s size, the H_act_ of an individual should scale physiologically with its functional attributes (Reich, 2000). Height directly indicates the position of the plants and their modules (trunks, branches, leaves) in the light hierarchy within a given community and is an essential determinant of a plants’ capacity of carbon gain (Sendall and Reich, 2013; Kenzo et al., 2015) and hydraulic transport (Ryan and Yoder, 1997; Koch et al., 2004). Our results based on a comprehensive individual-level-analysis were in agreement with the widely recognized fundamental trade-offs and allometric relations involving shifts with plant height in dry-mass allocation. The positive relationships of H_act_ with Ca, LA and Pd reflect the geometric allometry among whole-plant, crown and leaf dimensions for balancing the costs of mechanic support and resource acquisition (Ellsworth and Reich, 1993; Olson et al., 2009; Osada, 2011). With increasing H_act_, the enhancements of LA, LMA, LDMC, Amax, Lcp, Lex and Ca but the reductions in Ld and Lcov associate with the specific strategy of tall plants towards investing more photosynthetic carbon in constructing expensive but potentially long-lived leaves (i.e., great leaf mass per unit area) and in branches building a wide crown with a larger proportion of clumped leaves for intercepting ample sunlight (Horn, 1971; Milla and Reich, 2007). In contrast, light intensity declines from canopy to forest floor as incoming radiation is intercepted by tall plants (**Figure 4A**). As such, small woody understory plants, with their foliage positioned in a light-limited environment, cannot afford high construction costs with slow revenue of dry-mass per area; they produce low LMA leaves with relatively large light capturing area (Niinemets, 2007).

Interestingly, the relationships among H_act_, leaf physiological traits and hydraulic traits indicate that photosynthetic hydraulic limitation is also an important mechanism responsible for individual coexistence within a small local area. With an increase in water potential and transpiration flow needed to absorb water and transport it upward from understory to canopy, enhanced Amax, SD, Vd, E, Ks, Cond, Tr and Pd but reduced SM with increase in H_act_ (**Figure 5B**) suggest a strong hydraulic efficiency vs. safety trade-off among co-occurring plants. Tall plants being acquisitive in carbon gain (i.e., high leaf photosynthetic rate and stomatal density) are also acquisitive in water transport (i.e., great stomatal conductance, xylem vessel diameter, sapwood specific hydraulic conductivity and stem sap flow) and transpiration rates, at the expense of hydraulic safety (i.e., low stem hydraulic safety margin) (Schafer et al., 2000; Tyree and Zimmermann, 2002; Meinzer et al., 2009). For tall trees, the long path of water transportation inevitably causes a large risk of hydraulic failure due to strong hydraulic resistance or even cavitation; thereby, they have to manage high hydraulic conductance and/or efficiency (Ryan and Yoder, 1997; Prentice et al., 2014; Kenzo et al., 2015). Logically, the reverse is true for short plants in understory (McCulloh et al., 2015). In this study, such plant height-mediated hydraulic efficiency vs. safety trade-offs is also evident from the negative relationships of the proportion of dispersed leaves (Ld) with each of H_act_, Ca, Pd, LMA, Amax, Lex, SD, Vd, E, Lwp and Ks, and the positive LD-SM relationship. These contrasting patterns suggest that tall plants with a large proportion of clumped leaves are capable of gaining light and carbon efficiently, and moving water rapidly but unsafely. More clumped leaves per branch in a plant crown are beneficial not only for competing vertically for light but also for offsetting hydraulic resistance between terminal twigs and leaves (Poorter et al., 2003).

## 5 Conclusion

Overall, our results highlight the coordination of resource acquisition, mechanical support and hydraulic functions among locally interacting plants, promoting carbon gain and retention by coupling light capture and water management from trunk bottom to crown top. This study has important implications for understanding how variation in H_act_ influences strategic tradeoffs among co-occurring individuals at within-community scale. The within-community plant economic spectrum provides a useful framework for improving models that predict future vegetation based on individual-level variations in plant form and function. We encourage future trait-based studies focusing on the ecology of individuals instead of species to improve our understanding of plant ecological strategies, species coexistence and ecosystem functions such as carbon and water flux and storage.

## Data Availability

The raw data supporting the conclusions of this article will be made available by the authors, without undue reservation.

## Supplementary material

Supplementary Table 1 Characteristics of the studied species and individuals in *Schima superba* community in Tiantong National Forest Park, Zhejiang Province, China.

Supplementary Text 1 Detailed methods for trait measurement.

Supplementary Figure 1 Pearson correlation coefficients (r) between plant height, leaf and wood economic, physiological and hydraulic traits and crown architectural traits across woody species and/or across woody individuals at within-community scale.
