# Supplementary material for: Importance of the actual plant height in modulating the within-community spectrum of plant form and function
Source: Front Plant Sci. 2025 Jul 24;16:1616656. doi: 10.3389/fpls.2025.1616656 (PMC12328304; doi:10.3389/fpls.2025.1616656)

**Supporting Information.**

**Text A1.** Detailed methods for trait measurement

**Traits measured at both within-community and regional scales**

We measured mean leaf area (LA), leaf mass per area (LMA), leaf dry matter content (LDMC), leaf nitrogen content (Nmass) and wood density (WD) for individuals in both a *Schima superba* plot in the Tiantong National Forest Park and five sites across the region. In the field, we collected leaf, branch, and stem samples across individuals from July to September in 2015. We cut three large branches from a plant at its canopy rim, with at least ten fully-developed and healthy-looking leaves from each of the branches. The samples were wrapped with a moist paper towel and then stored in sealed plastic bags and kept cool until brought back to the laboratory for measurement, which was usually performed within 12 hrs of sample collection. In the laboratory, leaves and twigs were separated for each branch. Then the current year’s twig diameter at the basal point was measured using electronic vernier calipers, to the nearest 0.1 mm. Twig cross-sectional area was calculated from diameter assuming the twig to be approximately round in cross-section.

At the same time, 20 mature leaves per plant were combined from three branches and all fresh leaves attached to the current year’s twigs were each combined to form separate samples. The leaves in those samples were scanned using a leaf area meter (LI-3100C, Li-Cor, USA) to determine LA and total leaf area per twig. Then twig and leaf samples were dried at 75 °C in an oven for 72 hrs to determine twig and leaf dry mass, which were then used to calculate LMA (leaf dry mass divided by leaf area) and LDMC (fresh mass divided by dry mass). Subsequently, the leaf samples were ground to determine mass-based N concentration , which was measured using a discrete autoanalyzer (Smartchem 200, Alliance, France). Finally, remaining large branches were used to measure wood density (WD). The pith and bark were removed, and fresh volume was measured by water displacement. Then sample mass was oven-dried to a constant value at 105°C, and wood density was oven dry mass per fresh volume (g cm^-3^).

**Traits measured at within-community scale**

In addition to the above 6 traits collected both locally and regionally, 15 out of the 22 traits were specifically measured on the coexisting individuals in the *Schima superba* community in the Park. In the plot, H_act_ of each woody plant was measured with a telescope pole for heights up to 15 m, and with a Vertex meter (Vertex-IV, Haglöf, Dalarna, Sweden) for heights >15 m in July, 2015. Crown width for each plant was determined as the average diameter of the east–west and north–south cross sections of the crown using a meter stick. Crown area (Ca) was estimated by multiplying two diameters from two directions. Leaf coverage (Lcov), leaf convergence and crown light exposure index (Lex) of the tree crown were estimated by two independent observers in the field, and then the average value was used to reduce individual bias. Leaf coverage was estimated in 10 % cover classes. Leaf convergence was classified into clumped and dispersed group, respectively. Trees with the leaves being densely stacked in a single layer in the upper part of the crown were considered as clumped. In contrast, trees with leaves being stacked in several more widely spaced layers, and shading each other, were considered as dispersed (Poorter, Bongers & Bongers 2006). Here we used the proportion of dispersed leaves per crown (Ld, opposite to the proportion of clumped leaves assuming that the proportion of dispersed plus clumped leaves is 100 %) to quantitatively describe how plants acclimatize to light conditions and hydraulic restrictions in their leaf deployment per se. Lex was determined on a five-point scale for each plant: 1 = no direct light received in the crown area, 2 = lateral light received in the crown area, 3 = partial overhead light received in the crown area, 4 = more than 90% of the crown area receives direct overhead light, and 5 = emergent crown with direct light from all direction (Poorter, Bongers & Bongers 2006).

Physiological and hydraulic traits were measured during the plant growing season from July to September in each of 2014 and 2015. For each plant, one branch in the peripheral position (sunlit-side) of the crown was cut down and three leaves were selected specifically to measure petiole diameter (Pd) using vernier calipers in the laboratory. Although Pd is highly associated with plant physiological and hydraulic functions, we grouped them into architectural trait dimensions due to their morphological nature. Simultaneously, the sampled branch was quickly stored in a water filled bucket in the field. In order to avoid effects of blight or pests attack on leaves’ photosynthetic and transpiration activities , three healthy leaves per branch were selected for leaf photosynthetic and transpiration measurements. A light response curve for each leaf was measured between 8:00 am and 4:00 pm on each sunny day with a portable photosynthesis system (Li-6400XT, Li-Cor, USA). The gradient of photosynthetic photon flux density was set at 1600, 1200, 900, 600, 400, 300, 200, 150, 100, 75, 50, 25, and 0 μmol m^-2^ s^-1^ for the individuals with H_act_ larger than 8m, and at 1200, 900, 600, 400, 300, 200, 150, 100, 75, 50, 25, and 0 μmol m^-2^ ·s^-1^ for the individuals with Hact smaller than 8m, respectively. The light compensation point (Lcp) and the maximum photosynthetic rate (Amax) were calculated by photosynthesis assistant software (Dundee Scientific, Dundee, UK). Reported values about transpiration rate (Tr) and stomatal conductance (Cond) were measured when PPFD was 1500 mmol m^–2^ s^–1^. In addition, the remaining healthy leaves on the collected branches were also detached and saved in a refrigerator at 4°C for the measurement of stomatal density (SD).

In the laboratory, five fully expanded leaves per plant were painted with nail polish after any water droplets on the blade were absorbed using bibulous paper. Nail polish was smeared along the adaxial and abaxial leaf surface by avoiding major and secondary leaf veins (Volenikova & Ticha 2001) . Following parching up, nail polish was removed with tweezers. Then the leaf sample was mounted onto the stereoscopic microscope (SMZ-168, MOTIC, USA) for counting stomata number under a 0.20 mm^2^ window at x 40 magnification. SD was calculated as the ratio of the number of stomata to the total area of the measured window after scaling down 40 magnification.

To measure hydraulic and anatomical traits, three branches from each plant were harvested from the sun-exposed position of the plant crown using a long-reach pruner before the sun came out (i.e., predawn). These branch samples were sealed in a black plastic bag with a moist towel, and immediately transported to the laboratory within 15 min. Firstly, three healthy leaves were detached from each branch to measure leaf water potential (Lwp) using a pressure chamber (Model 1505D-EXP, PMS Instrument Company, Albany, OR, USA). We repeated this measurement three times for each leaf. We collected the 1-year-old twigs (3-6 mm in diameter) to measure hydraulic conductivity. In this process, a twig segment with length of 5-10 cm was re-cut from the sampled branch underwater and the attached leaves were separated. Prior to measurement, the distal ends of the twig segment were trimmed with a razor blade. By using the water perfusion method, hydraulic conductivity was measured by a high-pressure flow meter (HPFM-Gen3; Dynamax, USA) applied in a quasi-steady-state mode. Sapwood specific hydraulic conductivity (Ks, kg m^–1^ s^–1^ MPa^–1^) was equal to hydraulic conductivity divided by sapwood cross-section area. Sapwood cross-section area was calculated from twig diameter assuming the twig to be round in cross-section.

We also quantified the individual-specific stem hydraulic safety margin (SM) by measuring percent loss of hydraulic conductivity (PLC) under different stem xylem pressures. Vulnerability curves in branch samples were constructed using the air injection method (Cochard, Cruiziat & Tyree 1992). Briefly, a segment with a length of 5-7 cm was recut from two-year-old twigs for each sampled branch as described above and the segment ends were trimmed with a fresh razor blade under water. The segment was then attached to a hydraulic apparatus (XYL’EM-Plus, Bronkhorst, Montigny-les-Cormeilles, France) and perfused with degassed and distilled water to remove embolism until no bubbles spilled under a high pressure (0.2MPa), and then maximum hydraulic conductivity (K_max_) was measured. Subsequently, a pressure chamber (Model 1505D-EXP, PMS Instrument Company, Albany, OR, USA) was used to generate negative pressure in the xylem and provoke water-stress-induced cavitation. The pressure in the chamber was increased to 0.5 MPa and maintained for 15 min, after which the segment was transferred to XYL’EM-Plus to measure xylem hydraulic conductivity (K_i_). We repeated this process through increasing xylem pressure (P) by 0.5 or 1 MPa increments until more than 80% of the hydraulic conductivity was lost (often under 4 MPa). For each branch, two replicates were performed, thus there were six replicates for each plant. PLC was determined at each pressure step following the equation:

$PLC=100\times(1-\frac{K_{i}}{K_{max}})$ (1)

We produced vulnerability curves (VC) by plotting PLC against xylem pressure for each plant. VCs were fit with Weibull curves and bootstrapped 95% confidence intervals P_12_ and P_50_, which are respectively 12% and 50% loss of conductance, were extracted using the FITPLC package in R (Duursma & Choat 2017). P_12_ and P_50_ are physiologically significant indices because they are generally thought to respectively reflect the initial air-entry tension producing embolisms (Meinzer, Johnson, Lachenbruch, McCulloh & Woodruff 2009; Wason, Anstreicher, Stephansky, Huggett & Brodersen 2018) and the resistance to catastrophic xylem failure under extreme drought (Urli et al. 2013). The difference between P_12_ and P_50_ estimates the steepness of the vulnerability curve between these two key points. Here, we defined SM as P_12_-P_50_, which indicates a more gradual rise of PLC once xylem pressure has fallen below P_12_ (Meinzer, Johnson, Lachenbruch, McCulloh & Woodruff 2009). Mean values of embolism vulnerability parameters and SM correspond to the average values of six samples per plant.

Xylem vessel diameter (Vd) was measured for the same branches described above. Similarly, segments with a length of 5-7 cm were recut from two-year-old twigs for each branch and fixed in formal dehyde acetic-acid alcohol (FAA). Fixed twigs were cut on a sliding microtome and stained with safranin. Vessel lumen area and vessel density were determined from transverse twig sections of these segments, excluding pith. Images were taken using a microscope (Olympus DP73, Japan) fitted with a digital camera (QColor 3; Qimaging, Burnaby, BC, Canada). All branch samples were viewed at x 20 magnification, with vessels measured at lumen area of each vessel within a chosen sector bounded by rays (averaging 9.2928×10^-2^ mm^2^), adding sectors until at least 40 vessels had been measured (average 60 vessels per section). Vessel lumen areas were averaged to generate individual means; vessel diameter was calculated from the diameter of a circle of the given lumen area.

Stem sap flow was monitored using a Granier-type thermal dissipation probe (TDP) system for each plant during one year from July 2014 to July 2015, and subsequently the maximum of sap flow flux (E) in summer was used to characterize variation in water transportation property across plants. Two FLGS-TDP XM1000 systems (Dynamax Inc., Houston, TX, USA) were employed for monitoring sap flow across 60 individuals, since there were only 32 probes with TDP-30 mm needles in a single system. We installed TDP radially into the sapwood on the stem approximately 45 cm and 130 cm above ground for plants with Hact smaller and larger than 4 m, respectively, by avoiding bark scars on the stem area where holes were drilled and probes were inserted. To reduce the effects of compass direction with respect to sun exposure on variation in sap flow, we kept all probes toward the sun-exposed side on the stems across 60 individuals. Each probe consisted of two sensors, i.e., heated and unheated serving as a reference. To prevent thermal interference, we inserted the TDP needles with the heated needle into the top hole and the reference needle in the bottom hole, as recommended by the manufacturer. The sapwood-air interface around the needles of each probe was sealed with a plastic putty and surrounded with a waterproofing seal. This prevented water from touching the needle shaft, and causing a heat sink effect. The foam quarter-spheres were covered and taped on either side of the TDP needles to protect the sensor wiring from bending stresses, and to add thermal insulation around the needles. Finally, a reflective bubble wrap was wrapped around the tree, foam blocks, and the TDP probe installation.

Signals from the sensors were recorded every 30 min by a FLGS-TDP data logger for each day. The signal recorded was the temperature difference between the heated and unheated sensors that was dependent on the rate of sap flow around the probes. Sap flow rate (Js, g m^-2^ s^-1^) was calculated following equation 1 (Granier 1987).

$Js=119\times{(\frac{\Delta T_{m}-\Delta t}{\Delta T})}^{1.231}$ (2)

Where ΔT (°C) is the mean temperature difference between sensors during each half hour measurement interval and ΔT_m_ is the maximum ΔT when there is no sap flow.

Sap flow flux (E) was calculated as the product of Js by the sapwood cross-section area (As), i.e., E (g h^-1^) = As (cm^2^) × Js (g m^-2^ s^-1^) × 3.6. Sapwood cross-section area was estimated from its allometric relationship with the diameter at breast height (DHB) for a given species (Table S2). The allometric relationships of the studied species were obtained from cores taken from trees located nearby the sampled plot. Sapwood cross-section area was measured in 10 individuals with a wide range of DBH variation for a given species. In each of 19 species, a core was taken using an increment borer (5.15 mm diameter, Haglöf, Langsele, Sweden) at breast height from each of 10 plants. In the laboratory, sapwood thickness was manually measured using electronic vernier calipers, accurate to 0.1 mm, for each individual core; and the sapwood–heartwood boundary was generally easily made from the difference in colour or in transparency, due to differences in water content. Sapwood cross-section area was calculated from sapwood thickness by treating the sapwood cross-sectional area as a perfect circular shape. Finally, the allometric relationship between sapwood cross-sectional area and DBH of each species was fitted by an exponential function (Table S2).

Table A1 Regression equation of diameter at breast height (*DBH*) against sapwood

area (*As*) for the studied woody plant species in *Schima superba* community

| Species | Regression equation | *R*^2^ | *p*-value |
| --- | --- | --- | --- |
| *Camellia fraterna* | *A_s_* = 0.71 × *DBH*^1.67^ | 0.95 | < 0.001 |
| *Castanopsis carlesii* | *A_s_* = 0.64 × *DBH*^2.04^ | 0.99 | 0.007 |
| *Castanopsis fargesii* | *A_s_* = 1.33 × *DBH*^1.61^ | 0.99 | 0.01 |
| *Cyclobalanopsis glauca* | *A_s_* = 0.69 × *DBH*^2.04^ | 0.99 | 0.007 |
| *Cyclobalanopsis stewardiana* | *A_s_* = 1.02 × *DBH*^1.39^ | 0.99 | 0.01 |
| *Eurya loquaiana* | *A_s_* = 0.67 × *DBH*^1.83^ | 0.99 | 0.01 |
| *Eurya muricata* | *A_s_* = 0.73 × *DBH*^1.45^ | 0.97 | 0.03 |
| *Eurya rubiginosa* var.*attenuata* | *A_s_* = 0.26 × *DBH*^3.22^ | 0.99 | 0.01 |
| *Lithocarpus glaber* | *A_s_* = 1.05 × *DBH*^1.69^ | 0.99 | 0.01 |
| *Lithocarpus henryi* | *A_s_*=0.85 × *DBH*^1.83^ | 0.98 | 0.01 |
| *Loropetalum chinensis* | *A_s_* = 0.65 × *DBH*^1.97^ | 0.93 | 0.03 |
| *Machilus thunbergii* | *A_s_* =0.58 × *DBH*^1.84^ | 0.99 | 0.01 |
| *Neolitsea aurata* | *A_s_* = 0.58 × *DBH*^1.84^ | 0.99 | 0.01 |
| *Rhododendron ovatum* | *A_s_* = 0.45 × *DBH*^2.18^ | 0.96 | 0.006 |
| *Schima superba* | *A_s_* = 1.20 × *DBH*^1.64^ | 0.94 | < 0.001 |
| *Symplocos anomala* | *A_s_*=0.36 × *DBH*^2.24^ | 0.96 | 0.004 |
| *Symplocos caudate* | *A_s_* = 0.65 × *DBH*^1.86^ | 0.99 | < 0.001 |
| *Symplocos laurina* | *A_s_*=0.43 × *DBH*^2.13^ | 0.91 | 0.04 |
| *Symplocos stellaris* | *A_s_* = 0.57 × *DBH*^2.07^ | 0.96 | 0.002 |

**References:**

Cochard, H., Cruiziat, P. & Tyree, M.T. (1992) Use of positive pressures to establish vulnerability curves: further support for the air-seeding hypothesis and implications for pressure-volume analysis. *Plant Physiology,* **100,** 205-209.

Duursma, R. & Choat, B. (2017) fitplc - an R package to fit hydraulic vulnerability curves. *Journal of Plant Hydraulics,* **4,** 002.

Granier, A. (1987) Evaluation of transpiration in a Douglas-fir stand by means of sap flow measurements. *Tree Physiology,* **3,** 309-320.

Meinzer, F.C., Johnson, D.M., Lachenbruch, B., McCulloh, K.A. & Woodruff, D.R. (2009) Xylem hydraulic safety margins in woody plants: coordination of stomatal control of xylem tension with hydraulic capacitance. *Functional Ecology,* **23,** 922-930.

Poorter, L., Bongers, L. & Bongers, F. (2006) Architecture of 54 moist-forest tree species: Traits, trade-offs, and functional groups. *Ecology,* **87,** 1289-1301.

Urli, M., Porte, A.J., Cochard, H., Guengant, Y., Burlett, R. & Delzon, S. (2013) Xylem embolism threshold for catastrophic hydraulic failure in angiosperm trees. *Tree Physiology,* **33,** 672-683.

Volenikova, M. & Ticha, I. (2001) Insertion profiles in stomatal density and sizes in Nicotiana tabacum L. plantlets. *Biologia Plantarum,* **44,** 161-165.

Wason, J.W., Anstreicher, K.S., Stephansky, N., Huggett, B.A. & Brodersen, C.R. (2018) Hydraulic safety margins and air-seeding thresholds in roots, trunks, branches and petioles of four northern hardwood trees. *New Phytologist,* **219,** 77-88.

**Figure A1** Pearson correlation coefficients (r) between plant height, leaf and wood economic, physiological and hydraulic traits and crown architectural traits across woody species and/or across woody individuals at within-community scale. The top right part shows the species-level trait correlation, while the bottom left shows the individual level trait correlation. The shift of correlation coefficient from positive to negative is indicated by the color scaling from red to blue and the cross indicates an insignificant correlation (P >= 0.05). See Table 1 for trait acronyms.


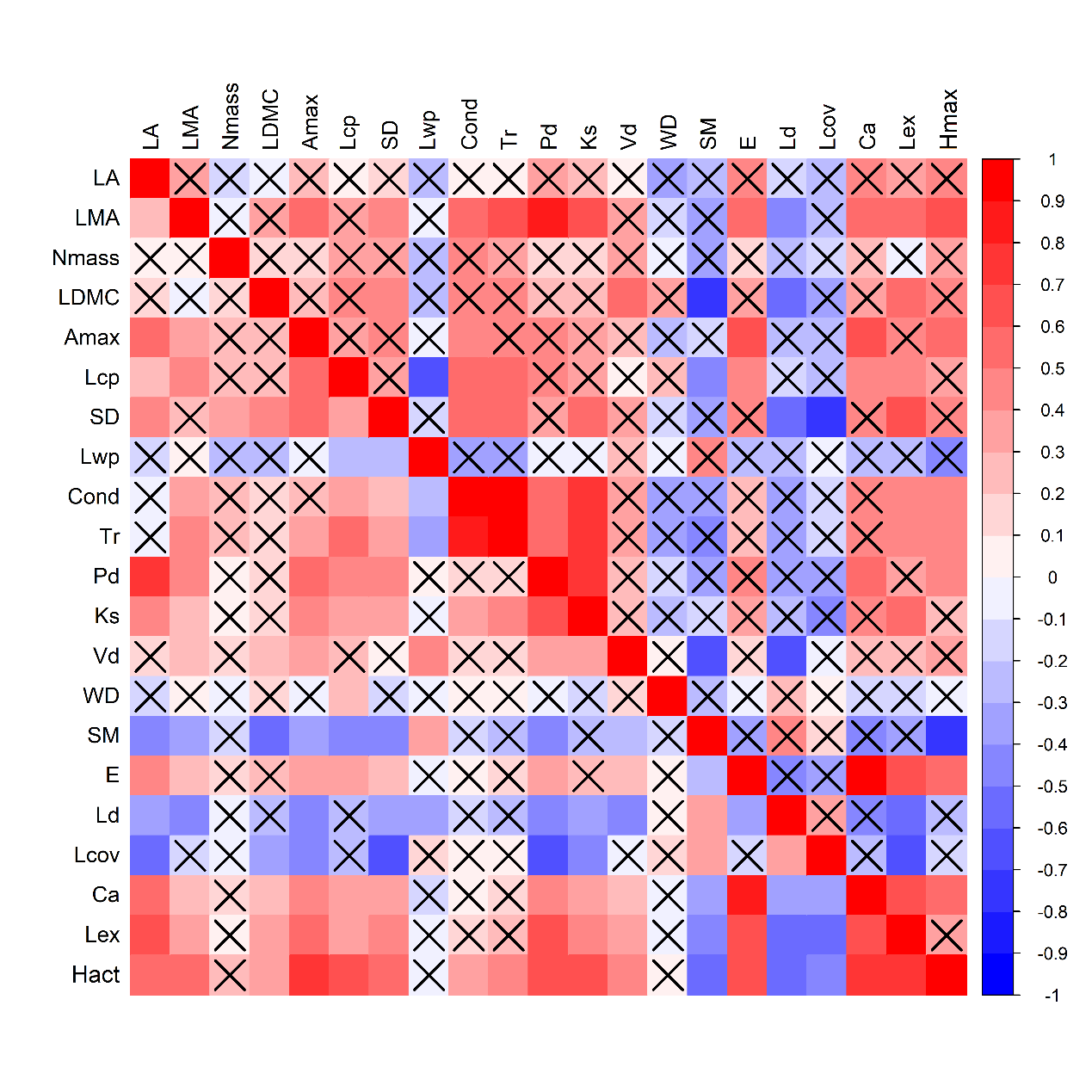

Supplement: Supplementary Table 1 — Characteristics of the studied species and individuals in Schima superba community in Tiantong National Forest Park, Zhejiang Province, China. [file SupplementaryFile1.docx]
